# Arranjos tecnológicos de gestão do cuidado em saúde: desafios para o hospital contemporâneo

**DOI:** 10.1590/0102-311XPT001025

**Published:** 2025-11-07

**Authors:** Arthur Chioro, Tiago Correia

**Affiliations:** 1 Universidade Federal de São Paulo, São Paulo, Brasil.; 2 Universidade Nova de Lisboa, Lisboa, Portugal.

**Keywords:** Assistência Integral a Saúde, Assistência Hospitalar, Assistência Centrada no Paciente, Relações Hospital-Paciente, Difusão de Inovações, Comprehensive Health Care, Hospital Care, Patient-Centered Care, Hospital-Patient Relations, Diffusion of Innovation, Atención Integral de Salud, Atención Hospitalaria, Atención Dirigida al Paciente, Relaciones Paciente-Hospital, Difusión de Innovaciones

## Abstract

O objetivo foi analisar arranjos tecnológicos na gestão do cuidado e de leitos em um hospital público de referência em Portugal. Arranjos tecnológicos combinam tecnologias e práticas de gestão para maior efetividade em contextos organizacionais complexos. São utilizados para enfrentar desafios como demanda excessiva, aumento de custos e queixas sobre qualidade do cuidado. Sua aplicação em distintos contextos têm produzido diferentes resultados e faltam estudos sobre seus impactos nas relações interprofissionais e na conexão entre hospitais e redes de serviços. Realizou-se estudo de caso qualitativo e etnográfico, envolvendo 15 entrevistas com 22 gerentes e trabalhadores e observação participante de três arranjos tecnológicos: o Serviço de Apoio à Gestão do Internamento, o Meeting das 11 Horas do Serviço de Medicina e o Grupo de Resolução High Users. Foram descritos arranjos tecnológicos para gestão de entrada (*input*), cuidado (*throughput*) e altas hospitalares (*output*). A observação em profundidade destacou a dinâmica, o potencial e os desafios dos arranjos tecnológicos para integrar uma gestão de leitos e altas. Quando bem implementados, os arranjos tecnológicos ajudam a enfrentar a superlotação hospitalar, mas não resolvem problemas sistêmicos de alta e continuidade de cuidado em rede. Evidenciou-se a forte adesão dos trabalhadores aos arranjos tecnológicos, com destaque ao protagonismo da enfermagem e o silêncio dos médicos, sugerindo novos desafios de autonomia e poder. Os arranjos tecnológicos demonstram efetividade na gestão de leitos e cuidado, alteram práticas multiprofissionais, e relações interprofissionais, além de afetar a rede de serviços externos. A produção do hospital contemporâneo requer arranjos tecnológicos baseados em evidências, que aumentem sua eficiência, sustentabilidade e capacidades técnica e organizacional.

## Abstract (en)

The aim of the present study was to analyze technological arrangements for the management of care and beds at a public reference hospital in Portugal. Technological arrangements combine technologies and management practices for greater effectiveness in complex organizational contexts and are used to address challenges, such as excessive demand, rising costs, and complaints with regards to the quality of care. The application of technological arrangements in different contexts has produced varying results and studies are needed to address the impact on interprofessional relationships and the connection between hospitals and service networks. A qualitative, ethnographic case study was conducted, involving 15 interviews with 22 administrators and employees as well as participant observation of three technological arrangements: Inpatient Management Support Service; 11 a.m. Meeting; and High Users Resolution Group. Technological arrangements for managing admissions (input), care (throughput), and hospital discharges (output) were described. The in-depth observation highlighted the dynamics, potential, and challenges of technological arrangements for integrating bed and discharge management. When well implemented, technological arrangements help address hospital overcrowding, but do not solve systemic problems related to discharge and the continuity of care in the network. Strong employee adherence to technological arrangements was found, with a focus on the leadership of nursing staff and the silence of physicians, suggesting new challenges related to autonomy and power. Technological arrangements are effective at bed and care management, alter multidisciplinary practices and interprofessional relationships, and exert an impact on the network of external services. The production of contemporary hospitals requires evidence-based technological arrangements that increase efficiency, sustainability, as well as technical and organizational capabilities.

## Abstract (es)

El objetivo del trabajo fue analizar disposiciones tecnológicas en la gestión de la atención y camas en un hospital público de referencia en Portugal. Las disposiciones tecnológicas combinan tecnologías y prácticas de gestión para mejorar la efectividad en contextos organizativos complejos. Se utilizan para hacer frente a retos como la demanda excesiva, el aumento de los costes y las quejas sobre la calidad de la atención. Su aplicación en distintos contextos ha producido resultados dispares y faltan estudios sobre sus impactos en las relaciones interprofesionales y en la conexión entre hospitales y redes de servicios. Se realizó un estudio de caso cualitativo y etnográfico, que incluyó 15 entrevistas a 22 gestores y trabajadores, así como observación participante de tres disposiciones tecnológicas: el Servicio de Apoyo a la Gestión de Ingresos, el Meeting de las 11 Horas y el Grupo de Resolución High Users. Se describieron disposiciones tecnológicas para la gestión de entrada (*input*), la atención (*throughput*) y las altas hospitalarias (*output*). La observación en profundidad destacó la dinámica, el potencial y los retos de los disposiciones tecnológicas para integrar la gestión de camas y altas. Cuando se implementan correctamente, los disposiciones tecnológicas ayudan a hacer frente al hacinamiento hospitalario, pero no solucionan los problemas sistémicos de las altas y la continuidad de la atención en red. Se evidenció la fuerte adhesión de los trabajadores a los disposiciones tecnológicas, destacando el protagonismo de la enfermería y el silencio de los médicos, lo que sugiere nuevos retos de autonomía y poder. Los disposiciones tecnológicas muestran su eficacia en la gestión de camas y cuidados, modifican las prácticas multiprofesionales y las relaciones interprofesionales, y afectan a la red de servicios externos. La producción del hospital contemporáneo requiere disposiciones tecnológicas basados en las evidencias, que aumenten su eficiencia, sostenibilidad y capacidades técnicasyorganizativas.

## Introdução

Arranjos tecnológicos de gestão do cuidado em saúde são modalidades de intervenção que aplicam conhecimento científico para aprimorar a gestão e produção do cuidado em contextos organizacionais complexos. Utilizam-se de tecnologias, práticas e instâncias de gestão, que, se preferencialmente combinados, promovem ganhos em efetividade assistencial e gerencial ^1^ ^,^ ^2^ ^,^ ^3^.

Os arranjos tecnológicos são utilizados em diferentes países e contextos sanitários, nos setores público e privado, com distintos resultados, configurações e graus de implementação ^4^ ^,^ ^5^. Procuram melhorar o desempenho organizacional e enfrentar desafios como demanda excessiva, aumento de custos, diminuição da efetividade do cuidado e queixas relativas à qualidade, refletidas em dilemas como superlotação, tempo excessivo de espera para atendimento ou indisponibilidade de leitos hospitalares para internações e procedimentos eletivos. A face mais visível do problema é a permanência indesejada de pacientes por longos períodos em macas nos corredores ou em Serviços Hospitalares de Emergência (SHE), que também é um importante fator estressor nas equipes ^6^ ^,^ ^7^ ^,^ ^8^.

Elementos teóricos e conceituais oriundos da Teoria Geral dos Sistemas fundamentam três classes de intervenções baseadas no uso de arranjos tecnológicos, destinadas: à entrada de pacientes (*input*), com arranjos tecnológicos dedicados às pressões exercidas por demanda excessiva nos serviços hospitalares; aos processos de cuidado (*throughput*), com intervenções voltadas ao aprimoramento dos processos de produção do cuidado e de gestão dos pacientes internados e a articulação do SHE com os demais serviços do hospital; e às saídas de pacientes (*output*), por meio de arranjos tecnológicos que visam prevenir reinternações e aprimorar a articulação do hospital com os demais componentes das redes de saúde para continuidade do cuidado ^9^ ^,^ ^10^ ^,^ ^11^.

Há décadas, hospitais públicos e privados ensaiam novos modos de qualificar e otimizar a gestão do cuidado ^12^. Em larga escala, adotam dispositivos que valorizam o trabalho em equipe, protocolizações e fazem uso intensivo de indicadores para acompanhamento e avaliação do desempenho ^13^ ^,^ ^14^. Temas afeitos e sensíveis para governança, compreendida como o estabelecimento das próprias regras de operação, dentro dos quais as instituições da sociedade, incluindo os hospitais, funcionam ^15^ ^,^ ^16^.

Todavia, pouco se tem abordado sobre a implementação, possíveis impactos e reconfigurações dos arranjos tecnológicos nas práticas de profissionais de saúde e nas relações do hospital com os demais serviços de saúde. Em particular, porque tais dispositivos formam um conjunto de elementos técnicos e ideológicos operados estrategicamente pelo poder.

O presente artigo busca, ao analisar como a implementação desses arranjos tecnológicos é vivenciada pelas equipes assistenciais e gerenciais em seus cotidianos, compreender as alterações produzidas nas práticas profissionais e nas relações intra e interprofissionais. Pretende, ainda, refletir como a operacionalização desses arranjos tecnológicos interferem nas relações entre o hospital e a rede de serviços de saúde, na perspectiva da integralidade e continuidade do cuidado.

## Metodologia

A investigação foi realizada por meio de uma pesquisa de caráter qualitativo, tipo estudo de caso ^17^, buscando aproximar o pesquisador do mundo do trabalho e do cuidado no hospital, compreendendo como ele é construído e experienciado pelas equipes, a partir de contribuições da etnografia ^18^. Interessava identificar tanto as linhas, circuitos, fluxogramas e mapas que percorrem a organização do cuidado, como também analisar as relações, interações, interdependências e estratégias que lhes dão substância e conteúdo humano.

A recusa da separação entre sujeito e objeto, entre pesquisador e pesquisado, imprimiu contornos mais participativos e interativos à investigação. Uma pesquisa realizada com os atores no campo de investigação, onde o ato de pesquisar foi sendo compartilhado e seus achados surgiram como substrato para a discussão do próprio trabalho e, portanto, como espaço de conhecimento ^19^. Procurou-se, ao mesmo tempo, assumir um rigor e uma vigilância epistemológica constante, estabelecendo um processo permanente de análise de implicação ^20^.

A pesquisa foi realizada entre maio e outubro de 2022, em uma unidade hospitalar pública de excelência, de grande porte e alta complexidade clínica e cirúrgica, que conta com serviços acreditados e é considerada um exemplo de gestão pública e de qualidade assistencial, entre maio e outubro de 2022. Trata-se de um hospital de referência para o Serviço Nacional de Saúde de Portugal, voltado também ao ensino, referência territorial para um distrito pertencente à Região de Saúde de Lisboa (Portugal).

As estratégias metodológicas escolhidas buscam uma aproximação micropolítica dos processos de gestão e de produção do cuidado e partem da aposta de que é necessário buscar referenciais teórico-conceituais e novas estratégias metodológicas, ancoradas no entendimento de que a saúde é uma construção humana e social. Uma maquinação de vários componentes entrelaçados. Da política, com seu alto grau de intencionalidades e extensividade. Da gestão, com suas prescrições, normas e controles. E da produção do cuidado em saúde, realizada a partir de múltiplas invenções cotidianas que os encontros singulares entre usuários e profissionais de saúde produzem, por dentro e por fora da política e da própria gestão. Para tanto, foram utilizadas estratégias metodológicas combinadas, descritas detalhadamente no Quadro 1.

**Quadro 1:** Estratégias metodológicas para produção e análise dos dados segundo objetivos específicos.

OBJETIVO	ESTRATÉGIA METODOLÓGICA	PRODUTOS OBTIDOS
Caracterizar os arranjos tecnológicos de gestão do cuidado operacionalizados no hospital estudado	Realização de 15 entrevistas individuais e em grupos com gestores e a equipe técnica do hospital, com a participação de 22 sujeitos	Mapeamento e caracterização dos arranjos tecnológicos operacionalizados no hospital estudado
Identificar e compreender como os arranjos tecnológicos são vivenciados pelas equipes dirigentes e assistenciais em seus cotidianos	Realização de entrevistas em profundidade e observação participante de três arranjos tecnológicos: Serviço de Apoio à Gestão do Internamento (SAGI): Meeting das 11 Horas do Serviço de Medicina; e Grupo de Resolução High Users (GRHU)	Diários de campo; Narrativas das equipes locais de observação; Caracterização em profundidade dos três arranjos tecnológicos selecionados
Analisar alterações nas relações entre o hospital e a rede de serviços de saúde na operacionalização desses arranjos tecnológicos, na perspectiva da integralidade e continuidade do cuidado		

Fonte: elaboração própria.

Em um primeiro plano analítico, visando a caracterização dos arranjos tecnológicos implementados no hospital de referência, foram realizadas 15 entrevistas, com 22 sujeitos, entre gestores e trabalhadores (Quadro 2).

**Quadro 2:** Entrevistas realizadas com gestores e trabalhadores do hospital de referência. Lisboa, Portugal, 2022.

ENTREVISTA	CARGO/FUNÇÃO
01	Administrador Hospitalar I
02	Chefe do Serviço de Ortopedia Administradora Hospitalar II Administradora Hospitalar III
03	Chefe do Serviço de Apoio à Gestão do Internamento (SAGI) Administradora Hospitalar IV Administrador Hospitalar V
04	Diretor do Serviço de Psiquiatria Administradora Hospitalar VI
05	Responsável pelo Grupo de Resolução High Users (GRHU) Administradora Hospitalar VII
06	Diretora do Serviço de Urgências Administradora Hospitalar VIII
07	Diretora do Serviço de Medicina Interna e da Equipe de Gestão de Altas
08	Enfermeira-chefe do Serviço de Medicina Interna e do SAGI
09	Médica responsável pela Unidade de Hospitalização Domiciliária (UHD) Chefe da Enfermagem da UHD
10	Responsável pela Unidade Local de Gestão do Acesso (ULGA)
11	Enfermeira da Equipe Técnica do GRHU
12	Enfermeira da Equipe Técnica de Gestão de Internações
13	Enfermeira da Equipe Técnica de Gestão de Altas
14	Diretora do Serviço Social
15	Administrador Hospitalar I

Fonte: elaboração própria.

Posteriormente, em um segundo plano, de maior profundidade analítica, realizou-se a observação participante de três arranjos tecnológicos: o Serviço de Apoio à Gestão do Internamento (SAGI), o Meeting das 11 Horas do Serviço de Medicina e o Grupo de Resolução High Users (GRHU). Para eleição de tais arranjos tecnológicos, levou-se em conta o que dirigentes e equipes entrevistadas reconheciam como apostas centrais e consideravam que a organização hospitalar tinha empreendido maiores investimentos e obtido melhores resultados visando as mudanças almejadas.

Procurou-se retratar cada um dos arranjos tecnológicos escolhidos nos seus componentes organizativos, formais e informais, a partir do plano das práticas cotidianas das equipes. Buscou-se identificar e analisar as transformações nas práticas e nas relações entre as várias categorias profissionais que atuam no hospital resultantes da operacionalização desses arranjos, assim como a relação destes com a rede de serviços.

Para realizar a observação, foi solicitada ao administrador hospitalar - ponto focal indicado pela direção do hospital, que intermediasse junto às chefias dos serviços onde os arranjos tecnológicos selecionados são implementados - a autorização para que o pesquisador pudesse acompanhar livremente o cotidiano do trabalho das equipes, participar de suas atividades e encontros formais, circular livremente pelos serviços e dialogar com usuários e trabalhadores.

Com a permissão para uma observação de caráter participante, pôde-se contar com a colaboração das equipes de gestão e assistência. As informações foram sendo produzidas e registradas em diário de campo, composto por registros de observações, percepções e narrativas sobre o funcionamento dos arranjos tecnológicos escolhidos, em suas dimensões materiais (recursos tecnológicos e humanos empregados) e imateriais (relações, sentidos, disputas etc.).

A pesquisa contou com bolsa de pesquisa no exterior financiada pela Coordenação de Aperfeiçoamento de Pessoal de Nível Superior (CAPES, Brasil) e foi autorizada pelo Conselho de Administração do hospital público português estudado e aprovada por seu Comitê de Investigação Científica (parecer C55822072912570). Foram observadas as exigências éticas para realização de pesquisas com seres humanos. Os participantes assinaram Termo de Consentimento Livre e Esclarecido (TCLE), garantindo-se o sigilo em relação às suas identidades, sem envolver outros riscos. As entrevistas foram gravadas em meio digital e transcritas.

## Resultados

### Os arranjos tecnológicos implementados no hospital

Os arranjos tecnológicos são implementados para atender necessidades práticas e urgentes, e buscam responder a problemas estruturais. Podem ser motivados por normas governamentais, propostas de agências de fomento, consultorias ou pela adaptação de experiências bem-sucedidas de outros serviços. Alguns lastreados em evidências que sustentam seus resultados. Outros, por modismos.

Os arranjos tecnológicos implementados destinam-se à gestão da entrada dos usuários, ao gerenciamento do cuidado e de leitos durante o internamento, e à produção de altas, continuidade do cuidado na rede de saúde e prevenção da reinternação (Quadros 3, 4 e 5, respectivamente).

**Quadro 3:** Arranjos tecnológicos destinados à gestão de entrada ou admissão dos usuários no estabelecimento (*input*).

SERVIÇO	ARRANJO	DESCRIÇÃO
Serviço de Urgência (SU)	Protocolo de Manchester	Triagem com classificação de risco efetuada pela enfermagem
	Protocolo para urgências de menor complexidade	Redirecionamento de casos classificados como “verdes” e “azuis” para Cuidados de Saúde Primários, com agendamento diário nas Unidades de Saúde da Família (USF), por meio de plataforma digital
	Vias Verdes	Linha de cuidado com fluxos próprios e acesso imediato para casos suspeitos de AVC, doenças coronárias e sépsis
	Grupo de Resolução High Users (GRHU)	Gestão do cuidado destinada especificamente a usuários hiperutilizadores do SU
	Escala de médicos da Medicina	Atribuição obrigatória de plantões para todos os médicos da Medicina no SU, com exceção para os dispensados legalmente
	Atribuição de casos aos especialistas	Atribuição de responsabilidade, para acompanhamento de pacientes internados no SU por falta de leitos, aos especialistas dos diferentes serviços do hospital
Conselho de Administração	Serviço de Apoio à Gestão do Internamento (SAGI)	Núcleo interno de regulação de leitos para gestão de internações e de altas hospitalares e preparação da transição de cuidados
Diretoria Clínica	Unidade Local de Gestão de Acesso (ULGA)	Gestão da lista interna e externa de espera cirúrgica (ambulatorial e internamento)
		Gestão da lista interna e externa de exames complementares
		Consulta de enfermagem pré-operatória
		Sistema Nacional de Lista de Espera Cirúrgica (LEC) - contratualização de unidades externas e *voucher* cirúrgico para suprir demanda excedente contratualizada
Serviço de Medicina	Hospital-dia Oncologia	Oferta de cuidados destinados à infusão de quimioterápicos e outros procedimentos que visam evitar a internação
	Projeto ICC e Tromboembolismo	Oferta de cuidados multiprofissionais em regime de hospital-dia que visam evitar a internação
Serviço de Psiquiatria	Consultoria semanal	Apoio matricial de especialistas em saúde mental aos médicos de família dos Centros de Saúde

AVC: acidente vascular cerebral; ICC: insuficiência cardíaca congestiva.

Fonte: elaboração própria.

**Quadro 4:** Arranjos tecnológicos destinados ao processamento da gestão do cuidado e dos leitos durante a fase de internamento (*throughput*).

SERVIÇO	ARRANJO	DESCRIÇÃO
Conselho de Administração	Serviço de Apoio à Gestão do Internamento (SAGI)	Núcleo interno de regulação de leitos para gestão de internações e altas hospitalares e preparação da transição de cuidados
Serviço de Medicina (SM)	Meeting das 11 Horas do Serviço de Medicina	Reuniões clínicas diárias com chefe, médico, enfermeiro e assistente social responsáveis por cada um dos SM, membros do SAGI e chefe do SU
	Quadro branco	Registro de informações sobre disponibilidades de leitos e providências necessárias, visando monitoramento coletivo da oferta de leitos e altas hospitalares
	Hospitalização Domiciliária	Oferta de equipes multidisciplinares dedicadas a 20 leitos de internação de pacientes em domicílio
	Hospital-dia	Indicação de pacientes para continuidade de cuidados em regime de hospital-dia
Diretoria Clínica	Leitos “flutuantes”	Internação dos pacientes em diferentes enfermarias, de acordo com a necessidade de leitos, sob cuidados da especialidade indicada para cada caso
	Unidade Local de Gestão de Acesso (ULGA)	Sistema Nacional de Lista de Espera Cirúrgica - contratualização de unidades externas e *voucher* cirúrgico
Serviço de Urgência (SU)	Triagem do doente ortopédico	Circuito informático para triagem de paciente
	Algoritmos cirúrgicos	Utilização de algoritmos para definição de pacientes com prioridade para resolução cirúrgica
	Reunião mensal com chefias de equipe	Reunião mensal com os chefes de todos os serviços, coordenada pelo SU
Serviço de Trauma-ortopedia	Médico clínico (hospitalista)	Designação de médico hospitalista para manejo clínico de pacientes idosos e com comorbidade
Gestão administrativa	Setorização da gestão	Atribuição de responsabilidades setoriais (administrativas, operacionais e assistenciais) aos administradores hospitalares

Fonte: elaboração própria.

**Quadro 5:** Arranjos tecnológicos destinados à produção de altas, continuidade dos cuidados e prevenção da reinternação (*output*).

SERVIÇO	ARRANJO	DESCRIÇÃO
Serviço de Medicina (SM)	Transição Segura	Carta de notificação de internações e altas destinadas aos médicos e enfermeiros das Unidades de Saúde da Família (USF)
		Reunião multiprofissional (enfermeiro e assistente social) para preparação de alta
		Reunião multiprofissional com as famílias de pacientes, com potencial de agravamento e dependência (48 horas após a internação)
	Hospitalização Domiciliária	Seleção e preparação de pacientes para regime de internação hospitalar em 20 leitos domiciliares
Conselho de Administração	Serviço de Apoio à Gestão do Internamento (SAGI)	Núcleo interno de regulação de leitos para gestão de internações e altas hospitalares e transição de cuidados
Serviço Social	Gabinete de transporte	Garantia de transporte sanitário imediato por meio adequado (táxi, ambulância ou meios próprios) para a residência ou instituição para o qual o paciente for encaminhado
	Contratualização de leitos	Contratação de vagas pelo hospital em instituições especializadas para idosos e em saúde mental para continuidade de cuidados e liberação de leitos
	Rede Nacional de Cuidados Continuados	Encaminhamento de pacientes para a Rede Nacional de Cuidados Continuados, de acordo com as normas estabelecidas pelo Ministério da Saúde

Fonte: elaboração própria.

Alguns arranjos implementados foram inspirados em práticas de outros países ou promovidos por consultorias e financiamento de organismos internacionais, como o SAGI, o Meeting das 11 Horas do Serviço de Medicina e as Vias Verdes. Outros são resultado de políticas nacionais de saúde, como o Sistema Nacional de Lista de Espera Cirúrgica e a contratação de serviços complementares. Iniciativas como o matriciamento psiquiátrico e a integração de cuidados continuados também abordam demandas sistêmicas.

Protocolos baseados em evidências e mapas de cuidados clínicos ajudam a melhorar a integração entre as equipes e garantem maior fluidez nos processos, promovendo a desospitalização segura e o acesso eficaz a exames e medicamentos ^21^. Esses arranjos reduzem a variabilidade das ações e aumentam a capacidade de gestão assistencial. Promovem, ainda, a desospitalização oportuna e segura, construída desde a internação até a transição segura de cuidados com o apoio de equipes assistenciais ^22^.

### A observação em profundidade de três arranjos tecnológicos

#### O SAGI

O SAGI foi criado em 2018 para centralizar a gestão de leitos no hospital e induzir a produção de altas precoces, otimizar sua utilização, reduzir a média de permanência e prevenir reinternações. Antes de sua criação, havia desigualdade na ocupação de leitos, com superlotação no Serviço de Urgências (SU) e leitos ociosos em outros setores.

Sua equipe, composta por médicos, enfermeiros, assistentes sociais e apoio administrativo, funciona de forma integrada, priorizando pacientes de acordo com as necessidades institucionais. O sistema facilita a alocação de pacientes, antecipação de altas e gerenciamento de transferências para a Rede Nacional de Cuidados Continuados ou redes externas.

Sua ação se inicia diariamente pela identificação de necessidades do SU e termina avaliando o impacto sobre a superlotação do SU e a necessária reprogramação de atividades.

O SAGI faz o hospital fluir. Pressiona diretores de serviços, médicos e a equipe multiprofissional a assumir responsabilidades pelo fluxo de pacientes, para que assumam a gestão do cuidado e não esqueçam pacientes nos leitos, à espera de interconsultas e realização de procedimentos diagnósticos ou terapêuticos. Provoca as equipes multiprofissionais para que busquem soluções otimizadas e desencadeiem os recursos necessários para a resolução das demandas de cada paciente.

A enfermagem ganha um papel estratégico, que se percebe como um reposicionamento de forças nas relações de poder, na coordenação dos processos, no registro e na checagem de providências. Os médicos percebem essa situação com certo desdém ou alívio. Sabem que serão cobrados, mas também que a enfermagem atuará naquilo que extrapole a dimensão clínica.

O SAGI fornece dados em tempo real sobre a ocupação e desempenho hospitalar, auxiliando na tomada de decisões e promovendo a eficiência. Ele também regula processos críticos, como uso de dispositivos médicos e materiais especiais, sendo essencial para o funcionamento do hospital.

Gerenciar as diferentes ofertas hospitalares segue sendo um desafio. As práticas habituais, o poder local e a rotina diária escondem como esses serviços são realmente usados, causando ineficiência e riscos para o paciente. Por isso, uma ferramenta eficaz para essa gestão, como os núcleos de regulação internos, é muito importante ^23^ ^,^ ^24^.

#### Meeting das 11 Horas do Serviço de Medicina

Esse arranjo foi apresentado como uma “novidade” destinada a enfrentar problemas de ocupação de leitos e produção de altas hospitalares. Implantado em 2020, como dispositivo para enfrentar a superlotação causada pela pandemia de COVID-19 no Serviço de Medicina (SM), que equivale no Brasil ao de clínica médica e especialidades clínicas, busca solucionar a espera por leitos nas enfermarias, agilizar altas das unidades de tratamento intensivo (UTI) e melhorar a qualidade do cuidado, resolvendo pendências como exames, transferências e problemas sociais de pacientes com alta clínica, mas que permanecem internados.

O arranjo, chamado majoritariamente de “reunião das 11”, reúne representantes das enfermarias, cuidadores intermediários, profissionais da atenção domiciliária, vinculadas ao SM, sendo composta majoritariamente por enfermeiros e assistentes sociais, além do administrador hospitalar e da coordenação do SAGI. Coordenado pela diretora médica do SM, inicia-se pontualmente às 11 horas e dura cerca de 15 minutos, com todos em pé ao redor de um quadro branco onde são registradas as informações sobre leitos, altas e pendências. A enfermeira chefe do SM ocupa posição de destaque, como responsável pelos registros no quadro branco e grande domínio sobre a situação dos pacientes.

Apesar do investimento em sistemas informatizados, a padronização dos registros é precária, e o quadro branco se torna essencial para a dinâmica. A informatização se apresenta como um fetiche. Há sempre a expectativa de que novos aplicativos e sistemas irão solucionar os problemas. A despeito da existência de um sistema de informatizado robusto, mantém-se registros não padronizados e informais.

Paradoxalmente, ainda que o registro no quadro branco seja muito confuso, o arranjo tecnológico funciona como elemento organizador do processo de trabalho da equipe, que lida com a informação de forma segura sobre a dinâmica de ocupação dos leitos, que o sistema informatizado não aporta. Pelo uso do quadro branco, ato contínuo, é que se dá a definição sobre quem será internado e onde será alocado, sempre uma difícil escolha.

A reunião ritualizada em torno do quadro branco compromete as equipes com a produção de altas e ajuda a aliviar a superlotação, além de interferir na gestão de demandas eletivas. Ela conecta-se a outras iniciativas, como hospitalização domiciliar e contratualização de leitos em lares, demonstrando a complexidade da micropolítica do hospital.

Ao final da sessão, a dispersão era sempre muito rápida e, em pequenos grupos, eram produzidos encaminhamentos sobre decisões acordadas. O coordenador médico e a enfermeira do SAGI definem o “plano de ocupação do hospital”, decidindo sobre transferências e internações. Às terças-feiras, ocorre também um encontro para tratar de casos com alta clínica definida, mas que permanecem internados por problemas sociais, como dependência de lares contratados, da rede nacional de cuidados continuados ou apoio da família. A dificuldade em liberar esses pacientes contribui significativamente para a superlotação do SU.

O *meeting* responde a uma dinâmica de relações micropolíticas que reforçam o *empowerment* do SM, conferindo a este um lugar de maior controle e visibilidade nas relações internas do hospital. Destaca-se, ainda, o protagonismo da enfermagem, que organiza informações estratégicas e assume um novo lugar de controle na organização, o que torna os enfermeiros mais imprescindíveis. Já os médicos parecem aceitar e valorizar esse papel, ainda que isso não reconfigure as relações de poder no interior do hospital. A alta clínica é o limite de seu engajamento, deixando questões práticas para outros profissionais.

O modelo tradicional de hospitais, centrado em visitas médicas e prescrições diárias, cede espaço a uma abordagem mais integrada e centrada no usuário. Ferramentas como o Meeting das 11 Horas do Serviço de Medicina, inspiradas no método Kanban ^25^, trazem resultados positivos por meio da gestão visual e pactuação coletiva. Esse modelo promove maior alinhamento entre gestores e equipes assistenciais, impactando o desempenho hospitalar, reduzindo conflitos e aprimorando o cuidado centrado no paciente.

O perfil dos pacientes é cada vez mais complexo e crítico, tornando necessárias novas relações de cuidado. O hospital contemporâneo demanda um outro tipo de profissional e de equipe de saúde. As apostas em gestão clínica colegiada permitem atravessar a fronteira da informação e progredir na direção de um cuidado centrado no usuário ^14^. Desse modo, dispositivos como *meetings*, *boards* ou outros espaços colegiados, que sejam genuinamente multiprofissionais e orientados à discussão de resultados e à construção coletiva de objetivos e processos de trabalho, geram um impacto positivo ^24^ ^,^ ^26^.

#### GRHU

O GRHU foi criado em 2016 pelo Conselho de Administração do hospital e pela diretoria de Cuidados Primários da Regional de Saúde. Após análise do uso do SU, verificou-se que os hiperutilizadores (HU) eram pessoas com transtornos psicossociais, doenças crônicas agudizadas e emergências oncológicas. Descobriu-se que os HU também utilizavam excessivamente a atenção primária e outros serviços especializados, fruto da inadequação e fragmentação dos planos de cuidados.

Para solucionar o problema, foi desenvolvida uma plataforma informática que identifica, monitora e registra os HU, comunicando informações entre equipes e orientando intervenções.

Em 2015, os HU representavam 8,6% dos pacientes, mas geravam 27,9% dos atendimentos no SU. Classificados como “muito frequentes” (> 10 consultas/ano no SU) e “frequentes” (> 4 consultas/ano no SU), esses pacientes passaram a ser analisados semanalmente por uma equipe multiprofissional que incluiu médicos, enfermeiros e assistentes sociais do hospital e dos centros de saúde. A equipe utilizou a figura do “gestor de casos” e estratégias personalizadas. Inicialmente, havia pacientes com até 76 consultas/ano, semelhante a hospitais estudados com perfil de uso similar ^27^. Hoje, o máximo é 12, e o número de HU caiu de mais de 200 para cerca de 90, com redução de 60% no uso do SU.

As intervenções incluem coleta de informações nos serviços, e com os pacientes, e elaboração de estratégias específicas para cada caso, registradas na plataforma. As reuniões semanais discutem até quatro casos, com foco em problemas clínicos ou sociais e na reavaliação de estratégias quando necessário. A padronização do sistema, embora criticada, facilita o monitoramento. O sucesso do GRHU rendeu prêmios ao hospital e replicações em outras unidades. A enfermagem exerce papel central, com maior adesão, satisfação e liderança na equipe.

## Discussão

### O que revela a adoção dos arranjos tecnológicos neste hospital?

A racionalidade instrumental dos arranjos tecnológicos expressa a influência da Nova Gestão Pública ^28^, que prioriza ideias de gestão das empresas privadas nos serviços públicos, partindo do pressuposto de que práticas empresariais melhoram tanto a eficiência quanto a eficácia das organizações públicas. Essa lógica, ainda que imperceptível para a maioria ou desejada por alguns dos entrevistados, está presente tanto em arranjos tecnológicos implementados por decisões internas da organização - como o SAGI e o Meeting das 11 Horas do Serviço de Medicina - como naqueles induzidos por políticas nacionais, como os sob responsabilidade da Unidade Local de Gestão de Acesso (ULGA).

Os arranjos tecnológicos que operam em dimensões sistêmicas do cuidado, como os encaminhamentos para a Rede Nacional de Cuidados Continuados, são essenciais para enfrentar desafios estruturais, como altas médias de permanência hospitalar e falta de destinos seguros para pacientes que não necessitam mais de cuidados agudos. Contudo, a implementação desses arranjos exige maior integração e flexibilidade para atender as especificidades locorregionais.

Outro ponto crítico é a internalização dos arranjos na prática cotidiana das equipes. Arranjos efetivos são aqueles que obtêm alta adesão dos profissionais e permitem organizações locais mais plásticas e responsivas ^29^.

Os bloqueios de linhas assistenciais devido à superutilização ou à falta de alternativas de cuidado ilustram a necessidade de redes parametrizadas e gerenciadas para garantir itinerários eficientes de entrada e saída ^30^. Além disso, problemas como altas tardias são associados a riscos clínicos, como infecções e complicações, e a custos adicionais significativos ^31^. Estudos também destacam o estresse sobre as equipes e as implicações para a qualidade do atendimento, reforçando a importância de desospitalização segura e planejada desde o início da internação, em articulação com as famílias e a rede de cuidados ^22^ ^,^ ^25^ ^,^ ^30^.

### Aprendizados a partir de três arranjos tecnológicos de referência

Considerando a partir daqui os três arranjos analisados, dois achados comuns merecem ser destacados.

Primeiro, ao forte protagonismo da enfermagem se sobrepõe um certo “silêncio dos médicos”, que advém de um momento muito singular no qual que eles vivem, divididos entre a defesa de uma posição historicamente consolidada no hospital, pelo menos desde os séculos XVIII e XIX, e a adesão às novas configurações de relações interprofissionais que, se por um lado são postas como inevitáveis e necessárias, por outro, parecem ameaçar de modo incontornável a autonomia e o poder de que desfrutaram até agora ^32^.

Outro achado, é a forte adesão dos trabalhadores aos arranjos estudados e ao exercício de um “olhar panóptico” sobre o que se passa nas enfermarias e nas entranhas do hospital, de modo que se espera que praticamente toda a equipe consiga ter uma visão de tudo o que se passa, em tempo real e em curtíssimas reuniões, geralmente conduzidas pela enfermagem ^29^.

De acordo com a definição contida na Política Nacional de Atenção Hospitalar do Brasil ^33^, de 2013, “*hospitais são instituições complexas, com densidade tecnológica específica, de caráter multiprofissional e interdisciplinar, responsáveis pela assistência aos usuários com condições agudas ou crônicas, que apresentem potencial de instabilização e de complicações de seu estado de saúde...*”.

Hospitais se tornaram locais de grande relevância e simbolismo social, com alta visibilidade e importância central na estrutura dos sistemas de saúde. Isso porque são espaços dedicados tanto ao cuidado quanto ao ensino e à formação de profissionais da saúde. Além disso, são ambientes onde profissionais de diversas áreas realizam seu trabalho, em meio a uma divisão técnico-especializada e social do trabalho e complexas relações de poder. Soma-se a isso o fato de responderem a inúmeros interesses corporativos e empresariais ligados ao complexo médico-industrial ^24^.

A definição da missão e do modo de funcionamento dos hospitais não foi um processo simples, sendo marcada por disputas e resistências ao longo do tempo. A atual organização da atenção hospitalar, seus desafios e problemas, está intrinsecamente conectada à evolução da medicina, da ciência e das tecnologias, assim como à conformação dos sistemas de saúde. Também é condicionada por decisões políticas, econômicas e sociais, pelo papel atribuído ao Estado, pelos interesses do mercado e pelo embate entre projetos e visões distintas sobre saúde e organizações de saúde ^24^ ^,^ ^34^.

Enfrentar e reverter a centralidade excessiva dos hospitais no sistema de saúde, bem como promover sua integração em rede, continuam sendo desafios cruciais. Mais do que simplesmente abrir novos hospitais ou ampliar leitos, é fundamental redefinir o papel e o perfil assistencial das instituições existentes. Isso implica inseri-las em redes de saúde, ao mesmo tempo em que se promovem profundas transformações em sua organização e gestão, a fim de que possam entregar o que delas se espera ^24^ ^,^ ^25^.

A recusa em aceitar essa mudança de paradigma gera consequências visíveis, como as frequentes reinternações e a ocorrência de eventos cardiovasculares graves em idades precoces. Outro reflexo são as sequelas evitáveis decorrentes de internações por causas externas, onde os pacientes não conseguem acesso ao cuidado necessário para uma reabilitação adequada ^24^ ^,^ ^31^ ^,^ ^34^.

O que mais se destaca, no entanto, é a percepção de desassistência, que surge da sensação de descaso quando os pacientes não conseguem o acesso que consideram necessário. Contudo, nem eles e nem seus familiares ficam inertes ^24^. Por meio do que chamamos de “agir leigo”, eles vão atrás do melhor cuidado que não lhes foi oferecido ^35^. Eles ignoram os fluxos e arranjos estabelecidos pelos gestores e refazem seus próprios itinerários terapêuticos para conseguir acesso à rede de serviços que julgam essencial ^24^ ^,^ ^36^.

Reconhecer o hospital como parte essencial e interligada à rede de saúde implica desenvolver planos de cuidado transversais. Nesses planos, a atenção primária à saúde é o ponto central, coordenando o cuidado do paciente em seu território e articulando-se com os demais serviços de saúde ^37^ ^,^ ^38^. O êxito dessa abordagem depende do uso adequado do adensamento tecnológico que o hospital oferece, de forma pontual e por tempo limitado, sempre integrado a planos terapêuticos individualizados. Isso garante aos pacientes a continuidade dos cuidados em toda a rede de saúde ^25^.

Essa abordagem cria um benefício mútuo. O hospital evita internações prolongadas e reinternações precoces, garantindo o cuidado fora de suas instalações e prevenindo agudizações graves de condições crônicas. O sistema de saúde se beneficia do uso mais eficiente dos recursos hospitalares de alta tecnologia, evita gastos desnecessários com exames e uso excessivo de serviços ambulatoriais e, ainda, reduz os riscos associados a internações prolongadas ^24^ ^,^ ^31^.

Tal mudança é necessária e urgente, mas não é fácil e tampouco natural. A introdução de arranjos tecnológicos gera resistências ao produzir mudanças na relação de poder. A readequação de rotinas e práticas proporcionam estranhamento e receios.

Segundo Haas et al. ^34^, a expansão da assistência pode ser um fator de risco para pacientes e profissionais. Isso acontece porque é difícil entender os novos processos e aplicar o conhecimento existente em um ambiente diferente. Da mesma forma, é o que se sucede com a implantação dos arranjos tecnológicos, que impõem mudanças de processos que enfrentam saberes e práticas arraigadas na dinâmica e no processo de trabalho hospitalar.

### Implicações para os hospitais enquanto organizações complexas

É crucial repensar como a atenção hospitalar é conduzida. O principal desafio é integrar os hospitais aos demais serviços da rede, criando planos de cuidado personalizados. Isso ajuda a reduzir o desperdício causado por serviços duplicados, resultados insatisfatórios devido a internações prolongadas desnecessárias ou pelo uso de intervenções caras e de alto risco ^24^. Os hospitais estão no “extremo tecnológico da escala”, e os cuidados se acumulam ali para atender às crescentes expectativas, a um custo crescente ^29^.

Hospitais contemporâneos precisam de projetos mais científicos e reguladores, capazes de cuidar e produzir benefícios para gestores, trabalhadores e, em especial, seus usuários, para que resultem em maior previsibilidade e sustentabilidade. Desta forma, precisam ser cada vez mais eficientes e desenvolverem capacidades organizacionais, técnicas, científicas e econômicas. No entanto, essa abordagem racionalizadora, que se desenvolveu no setor privado sob a lógica do mercado (com foco em competitividade, redução de custos e lucro), frequentemente se distorce ao ser aplicada no setor público. Isso ocorre porque é inadequada para a gestão de organizações que não visam apenas a produção de valor. Essas instituições públicas demandam práticas gerenciais mais complexas para que o trabalho em saúde adquira outros significados ^24^ ^,^ ^34^.

A gestão hospitalar lida com a crescente racionalização das práticas hospitalares, de forma que estes estabelecimentos, públicos e privados, estão cada vez mais semelhantes, transformando-se em “hospitais-empresa”. Eles se integram à economia global e são constantemente avaliados por custo-benefício. Como Carapinheiro ^36^ já apontava, são peças-chave do complexo médico-industrial, influenciados por estratégias das indústrias de medicamentos e equipamentos. As decisões vitais para o hospital são frequentemente tomadas em instâncias externas. Além disso, enfrentam a automação e informatização crescentes, a terceirização de serviços e a mudança da relação médico-paciente para médico-organização. Isso gera uma especialização cada vez maior da prática médica, hierarquizada entre especialidades e com novas interdependências técnicas e funcionais ^12^ ^,^ ^24^.

Produzir um hospital com papel estratégico na oferta de cuidado é um desafio crescente. É crucial disputar esse espaço para transformar o modelo assistencial e criar novas formas de cuidado. Embora a busca pelo melhor processo assistencial e administrativo seja um discurso comum entre gestores hospitalares, isso, por si só, não garante a qualidade ^24^ ^,^ ^26^. Não basta adotar abordagens como o Lean, amplamente difundido em contextos hospitalares globais. Ainda que reduzam a média de permanência hospitalar, apresentam resultados limitados na satisfação de pacientes e equipes, e não se tem consenso sobre seus impactos positivos quando introduzidos no setor de saúde ^13^ ^,^ ^39^. Menos, ainda, integrar diferentes estratégias ^40^, em busca de agilidade e competitividade, tratando o hospital como uma mera empresa, com valor de uso para processos de apoio administrativo mais estruturados, mas que não respondem às questões diretamente envolvidas com no cuidado.

A qualidade, enquanto conceito, abriga algo de subjetivo e por isso exige padrões comparativos como balizadores de limites aceitáveis do que se considera “melhor e com mais qualidade” ^37^. É esperado que padrões de qualidade tenham graus diferentes de sucesso de acordo com o tempo e esforço de implantação e estabeleçam “valor de mercado” ^41^. A ênfase em processos de acreditação se dá às custas do citado valor de mercado. A busca por um selo de acreditação se tornou, por vezes, a razão em si do esforço, ao invés de traduzir o sucesso na implantação de transformações almejadas.

Além de ter uma organização hospitalar bem estruturada e acreditada, focada em resultados, surge a oportunidade de desenvolver práticas que coloquem o usuário-cidadão no centro do cuidado. Isso significa planejar a governança, a gestão clínica e a gestão de leitos sob a perspectiva da integralidade ^42^. Essa transformação só será possível se o hospital estiver fortemente comprometido com o cuidado integral e inserido em uma rede de cuidados. É preciso ir além da dimensão meramente técnica ou burocrática imposta pela lógica dos especialistas, equipes profissionais e gestores ^24^ ^,^ ^26^.

## Considerações finais

Há diversas intervenções que utilizam tecnologias para melhorar a gestão do cuidado e do uso de leitos hospitalares. Analisá-las pode ajudar a entender a relação entre a gestão hospitalar e o cuidado em rede, além de avaliar o que ocorre com os pacientes após a alta e os mecanismos de regulação para o cuidado pós-hospitalar.

A reestruturação de sistemas de saúde e da atenção hospitalar sem mudanças nas práticas de cuidado dentro do hospital é limitada. O caso estudado é um claro exemplo. Processos que focam apenas na racionalização do hospital não são suficientes para construir um hospital contemporâneo. Arranjos tecnológicos de caráter multiprofissional podem melhorar a entrada do paciente, a gestão clínica e gerar altas mais articuladas com outros pontos da rede, além de reduzir a superlotação, quando combinadas com outras intervenções.

A ampliação de leitos não resolve o problema de permanência prolongada. Já os arranjos tecnológicos focados na gestão do cuidado e leitos são mais efetivos para reduzir a superlotação. A produção de inovações na gestão hospitalar pode aprimorar as políticas públicas de saúde, embora mudanças disruptivas sejam menos impactantes na saúde que em outros setores.

Arranjos tecnológicos indicam novos formatos de relações interprofissionais, mas não alteram profundamente as relações de poder, especialmente com os médicos. É necessário expandir o estudo sobre arranjos tecnológicos para superar modelos simplificados e compreender melhor as dinâmicas organizacionais e os desafios das redes de cuidado.

A pesquisa realizada em Portugal ajudou a aprofundar a compreensão das complexas relações entre gestão e cuidado hospitalar. A produção de atenção hospitalar, alinhada aos desafios contemporâneos, deve ser parte da agenda estratégica para a resiliência dos sistemas de saúde, com o hospital sendo um espaço de inovação e de cuidado para os pacientes, profissionais e gestores.

## Data Availability

Os dados de pesquisa estão disponíveis mediante solicitação à autora de correspondência.

## References

[1] Dussauge P, Hart S, Ramanantsoa B (1992). Strategic technology management: integrating product technology into global business strategies for the 1990s.

[2] Bittencourt RJ, Hortale VA (2009). Intervenções para solucionar a superlotação nos serviços de emergência hospitalar: uma revisão sistemática.. Cad Saúde Publica.

[3] Crane J, Noon C (2011). The definitive guide to emergency department operational improvement.

[4] Wang M, Wild S, Hilfiker G, Chmiel C, Sidler P, Eichler K (2014). Hospital-integrated general practice a promising way to manage walk-in patients in emergency departments. J Eval Clin Pract.

[5] Beltrammi DGM (2015). Efetividade das intervenções para redução da superlotação nos serviços de emergência hospitalar.

[6] Weiss SJ, Derlet R, Arndahl J, Ernst AA, Richards J, Fernández-Frackelton M (2004). Estimating the degree of emergency department overcrowding in academic medical centers: results of the National ED Overcrowding Study (NEDOCS).. Acad Emerg Med.

[7] Magid DJ, Asplin BR, Wears RL (2004). The quality gap searching for the consequences of emergency department crowding. Ann Emerg Med.

[8] Cecilio LCO, Chioro dos Reis AA, Andreazza R, Spedo SM, Cruz NLM, Barros LS, Carapinheiro G (2020). Nurses in the Kanban: are there news meanings of professional practice in innovative tools for hospital care management?. Ciênc Saúde Colet.

[9] Donabedian A, Donabedian A (2003). The definition of quality and approaches to its assessment.

[10] Bradley VM (2005). Placing emergency department crowding on the decision agenda. J Emerg Nurs.

[11] Liu SW, Singer SJ, Sun BC, Camargo CA (2011). A conceptual model for assessing quality of care for patients boarding in the emergency department structure-process-outcome. Acad Emerg Med.

[12] Carapinheiro G (1993). Saberes e poderes no hospital. Uma sociologia dos serviços hospitalares..

[13] D'Andreamatteo A, Ianni L, Lega F, Sargiacomo M (2015). Lean in healthcare: a comprehensive review.. Health Policy.

[14] Campos GWS, Amaral MA (2007). A clínica ampliada e compartilhada, a gestão democrática e redes de atenção como referenciais teórico-operacionais para a reforma do hospital.. Ciênc Saúde Colet.

[15] Durán A, Saltman R, Durán A, Wright S (2020). Understanding hospitals in changing health systems.

[16] Akmal A, Greatbanks R, Foote J (2020). Lean thinking in healthcare findings from a systematic literature network and bibliometric analysis. Health Policy.

[17] Albarello L, Digneffes F, Hiernaux JP, Maroy C, Ruquoy D, Saint-Georges P (2000). Práticas e métodos de investigação em ciências sociais.

[18] Correia T (2012). Medicina. O agir numa saúde em mudança.

[19] Passos E, Barros RB, Passos E, Kastrup V, Tedesco S (2014). Pistas do método cartográfico: a experiência da pesquisa e o plano do comum.

[20] Altoé S (2004). René Lourau: analista institucional em tempo integral.

[21] McLachlan S, Kyrimi E, Dube K, Fenton N (2019). Proceedings of the 12th International Joint Conference on Biomedical Systems and Technologies.

[22] Andreazza R, Chioro A, Bragagnolo LM, Silva FF, Pereira AL, Mauri L (2023). Planned discharge and the inter-professional relationship from the perspective of the nursing actions during the COVID-19 pandemic.. Cienc. Saúde Colet.

[23] Vega RC, Pinto GO, Ribeiro FF, Spiegel T (2022). Pull processes in health care a systematic literature review. Gestão & Produção.

[24] Chioro A, Furtado LAC, Beltrammi DGM, Souza MP, Santos TBS, Pinto ICM (2021). Gestão hospitalar no SUS.

[25] Oliveira IS, Lima EFA, Silva RIC, Crozeta K, Dias ICB, Primo CC (2020). Beds management in the urgency and emergency using kanban. Res Soc Dev.

[26] Beltrammi DGM, Camargo VM (2017). Práticas e saberes no hospital contemporâneo: o novo normal.

[27] Sacoman TM, Beltrammi DGM, Andreazza R, Cecilio LCO, Chioro A (2019). Implantação do Sistema de Classificação de Risco Manchester em uma rede municipal de urgência. Saúde Debate.

[28] Osborne SP (2010). The new public governance?.

[29] Cecílio LCO, Chioro A, Andreazza R, Araujo EC, Schveitzer MC, Gomes A (2018). 17th ESHMS Biennial Conference: Old Tensions, Emerging Paradoxes in Health Rigths, Knowledge, and Trust. Book of Abstracts..

[30] Durán A, Wright S, Durán A, Wright S (2020). Understanding hospitals in changing health systems.

[31] Grabois V, Bittencourt R, Sousa P, Mendes W (2019). Segurança do paciente: conhecendo os riscos nas organizações de saúde..

[32] Cecílio LCO, Chioro A, Souza ALM, Cruz NLM, Spedo SM, Bizetto OF (2018). 17th ESHMS Biennial Conference: Old Tensions, Emerging Paradoxes in Health Rigths, Knowledge, and Trust. Book of Abstracts..

[33] Ministério da Saúde (2013). Portaria nº 3.390, de 30 de dezembro de 2013. Institui a Política Nacional de Atenção Hospitalar (PNHOSP) no âmbito do Sistema Único de Saúde (SUS), estabelecendo- se as diretrizes para a organização do componente hospitalar da Rede de Atenção à Saúde (RAS).. Diário Oficial da União.

[34] Haas SAG, Gawande A, Reynolds ME (2018). The risks to patient safety from health system expansions. JAMA.

[35] Cecilio LCO, Carapinheiro C, Andreazza R, Souza ALM, Andrade MGG, Santiago SM (2014). O agir leigo e o cuidado em saúde a produção de mapas de cuidado. Cad Saúde Pública.

[36] Carapinheiro G (2001). Inventar percursos, reinventar realidades doentes, trajetórias sociais e realidades formais. Etnográfica.

[37] Cecilio LCO, Reis AAC (2018). Apontamentos sobre os desafios (ainda) atuais da atenção básica à saúde.. Cad Saúde Pública.

[38] Allder S, Silvester K, Walley P (2010). Managing capacity and demand across the patient journey. Clin Med (London).

[39] Evans JM, Grudniewicz A, Baker GR, Wodchis WP (2016). Organizational capabilities for integrating care a review of measurement tools. Eval Health Prof.

[40] Zepeda-Lugo C, Tlapa D, Baez-Lopez Y, Limon-Romero J, Ontiveros S, Perez-Sanchez A (2020). Assessing the impact of lean healthcare on inpatient care a systematic review. Int J Environ Res Public Health.

[41] Ahmed W (2022). Understanding alignment between lean and agile strategies using Triple-A model. International Journal of Productivity and Performance Management.

[42] Cecilio LCO, Merhy EE, Pinheiro R, Mattos RA (2003). Construção da integralidade: cotidiano, saberes e práticas em saúde.

